# Supplementary material for: Unfolding the secrets of microbiome (Symbiodiniaceae and bacteria) in cold-water coral
Source: Microbiol Spectr. 2023 Sep 20;11(5):e01315-23. doi: 10.1128/spectrum.01315-23 (PMC10580923; doi:10.1128/spectrum.01315-23)
Supplement: Legends of supplemental files — Descriptions of supplemental files 1 to 5. [file spectrum.01315-23-s0001.docx]

**DESCRIPTIONS OF SUPPLEMENTAL FILES**

**Supplemental file 1.** List of actively transcribed genes of host.

**Supplemental file 2.** List of actively transcribed genes of Symbiodiniaceae.

**Supplemental file 3.** List of actively transcribed genes of bacteria.

**Supplemental file 4.** Physical and chemical conditions for each sampling site.

**Supplemental file 5.** Sequences of *COI* gene.
