# Supplementary material for: Unfolding the secrets of microbiome (Symbiodiniaceae and bacteria) in cold-water coral
Source: Microbiol Spectr. 2023 Sep 20;11(5):e01315-23. doi: 10.1128/spectrum.01315-23 (PMC10580923; doi:10.1128/spectrum.01315-23)
Supplement: Fig. S1 — Phylogenetic trees of new candidate bacteria phylotypes. [file spectrum.01315-23-s0002.docx]

**Supplementary file for:**

Unfolding the Secrets of Microbiome (Symbiodiniaceae and Bacteria) In Cold-water Coral

Sanqiang Gong^a,b^, Jiayuan Liang^b^, Xujie Jin^a^, Lijia Xu^c^, Meixia Zhao^a*^, Kefu Yu^b*^

^a^Key Laboratory of Tropical Marine Bio-resources and Ecology & Guangdong Provincial Key Laboratory of Applied Marine Biology, South China Sea Institute of Oceanology, Chinese Academy of Sciences, Guangzhou, China

^b^Coral Reef Research Center of China, Guangxi University, Nanning, 53004, China

^c^South China Institute of Environmental Sciences, The Ministry of Ecology and Environment of PRC, Guangzhou, 510530, China

*****Corresponding author:

Meixia Zhao

TEL: 020-89023342

E-mail: zhaomeix@scsio.ac.cn

Kefu Yu

Email: kefuyu@scsio.ac.cn

**
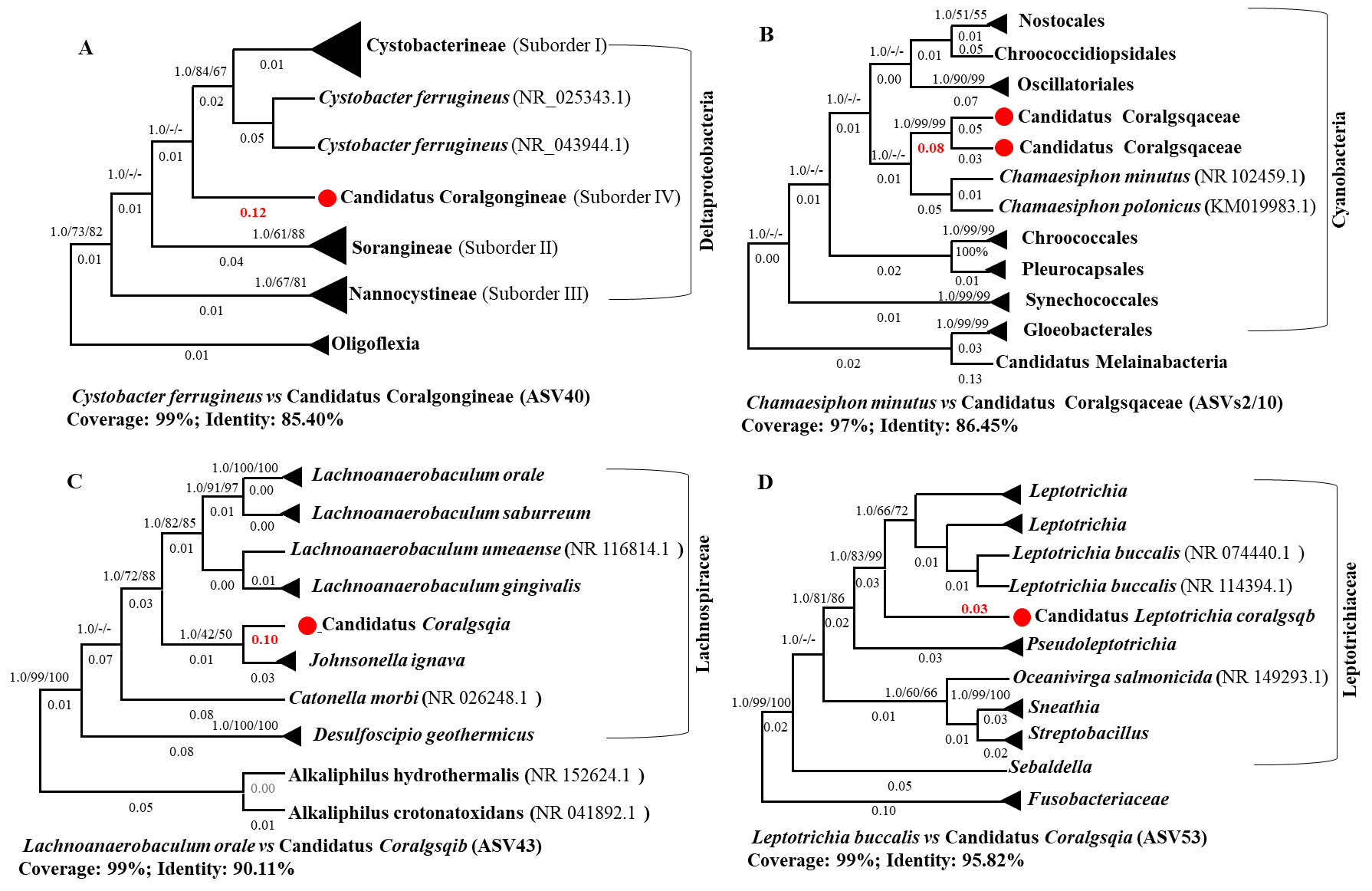
**

**FIG S1A-S1D. Phylogeny of new candidate bacteria phyla. Phylogenetic trees based on 16S rRNA gene sequences derived from our work and reference sequences from NCBI (https://www.ncbi.nlm.nih.gov/). Support values at the nodes represent Bayesian posterior probabilities, ML/NJ bootstrap support.**
